# One-Step Fabrication of Stimuli-Responsive Chitosan-Platinum Brushes for *Listeria monocytogenes* Detection

**DOI:** 10.3390/bios11120511

**Published:** 2021-12-13

**Authors:** Daniela A. Oliveira, Suleiman Althawab, Eric S. McLamore, Carmen L. Gomes

**Affiliations:** 1 Department of Biological and Agricultural Engineering, Texas A&M University, College Station, TX 77843, USA; daoliveira@tamu.edu (D.A.O.); suleimanth@tamu.edu (S.A.); 2 Department of Nutrition and Food Science, Texas A&M University, College Station, TX 77843, USA; 3 Department of Agricultural Sciences, Clemson University, Clemson, SC 26631, USA; 4 Department of Mechanical Engineering, Iowa State University, Ames, IA 50011, USA

**Keywords:** *Listeria* spp., biosensor, brush actuation, pH-responsive polymer, sonoelectrodeposition, foodborne pathogens, food safety

## Abstract

Bacterial contamination in food-processing facilities is a critical issue that leads to outbreaks compromising the integrity of the food supply and public health. We developed a label-free and rapid electrochemical biosensor for *Listeria monocytogenes* detection using a new one-step simultaneous sonoelectrodeposition of platinum and chitosan (CHI/Pt) to create a biomimetic nanostructure that actuates under pH changes. The XPS analysis shows the effective co-deposition of chitosan and platinum on the electrode surface. This deposition was optimized to enhance the electroactive surface area by 11 times compared with a bare platinum–iridium electrode (*p* < 0.05). Electrochemical behavior during chitosan actuation (pH-stimulated osmotic swelling) was characterized with three different redox probes (positive, neutral, and negative charge) above and below the isoelectric point of chitosan. These results showed that using a negatively charged redox probe led to the highest electroactive surface area, corroborating previous studies of stimulus–response polymers on metal electrodes. Following this material characterization, CHI/Pt brushes were functionalized with aptamers selective for *L. monocytogenes* capture. These aptasensors were functional at concentrations up to 10^6^ CFU/mL with no preconcentration nor extraneous reagent addition. Selectivity was assessed in the presence of other Gram-positive bacteria (*Staphylococcus aureus*) and with a food product (chicken broth). Actuation led to improved *L. monocytogenes* detection with a low limit of detection (33 CFU/10 mL in chicken broth). The aptasensor developed herein offers a simple fabrication procedure with only one-step deposition followed by functionalization and rapid *L. monocytogenes* detection, with 15 min bacteria capture and 2 min sensing.

## 1. Introduction

Incidents involving foodborne infection continue to be an important concern to consumers and a significant financial and marketing burden for the food manufacturers, regardless of the efforts to prevent them. The Centers for Disease Control and Prevention (CDC) estimates that each year in the U.S., about 1 in 6 people (or 48 million) become sick, 128,000 are hospitalized, and 3000 die from a foodborne illness. *Listeria* spp. is estimated to be in third place on the overall rank of pathogens contributing to domestically acquired foodborne illnesses resulting in death after *Salmonella enterica* and *Toxoplasma gondii* [1]. In 2020, the US Food and Drug Administration (FDA) reported 50 product recalls due to potential contamination with *Listeria* spp. [2]. Because of the severity of *Listeria* infection, the prevalence of this bacteria in food products, including fresh produce, ready-to-eat, refrigerated, and frozen products, it is important to find detection methods that are faster than the currently available, such as culturing the bacteria from samples which usually require pre-enrichment steps. The traditional methods used to detect foodborne pathogens in the food industry—plate counts, enzyme-linked immunosorbent assay (ELISA), and polymerase chain reaction (PCR)—are laborious, time consuming, and expensive [3]. The Food Safety and Modernization Act (FSMA), launched by the FDA in 2011, was designed as a proactive rather than a reactionary approach to improving public health by focusing on preventing food pathogen outbreaks. FSMA mandates comprehensive, science-based preventive controls compulsory for all food products [4]. Hence, the development of biosensing platforms has become a trending area of research for bacteria detection, as these technologies can lead to reliable and rapid screening unattainable through conventional techniques. The outcome of such screening aims to determine whether foods are contaminated with pathogens in time to prevent them from reaching the public or contributing to food waste [5,6].

Electrochemical biosensors can detect targeted bacteria by sensing the changes in electrical properties caused by biochemical reactions or molecular interactions at the surface of the working electrode [7,8]. Among the different possible biorecognition agents (including enzymes and antibodies), several aptamers (oligonucleotide sequences identified through selective evolutionary enrichment) and aptamer-based sensor platforms (aptasensors) have been developed to detect cell surface targets on intact *Listeria monocytogenes* [7]. Aptamers show similar or higher affinity for whole-cell targets than monoclonal antibodies and have the advantage of being smaller and allowing higher densities of pathogen-sensing elements leading to higher sensitivity and lower nonspecific adsorption [8]. Additionally, aptamers present better chemical stability under most environmental conditions and longer shelf life than antibodies [7]. Aptasensors have shown promising results for bacteria sensing, as recently reviewed with fast response time (detection in minutes to hours), no pre-enrichment requirements, and detection limits ranging from 10 to 10^3^ CFU/mL [8].

Platinum, deposited as nanoparticles, has been used in sensing as a signal amplification strategy. It improves sensors’ performance due to higher current densities and faster mass transport compared to larger macroparticles, as well as increased electrocatalytic behavior [9]. Electrodeposition is a versatile and efficient process in which an imposed electric field is employed to direct charged particles dispersed in a liquid towards an electrode for the assembly of thin films [10]; however, the use of standard potentiostatic or galvanostatic methods produces an array of nanoparticle sizes and morphologies formed due to the progressive generation of nucleation sites, rather than instantaneous formation [9]. Taguchi et al. [9] developed pulSED, a method combining pulsing electrodeposition and sonication that provided more uniform deposition of platinum nanostructures resulting in increased mass transfer to the electrode surface. 

Chitosan is a natural biodegradable, biocompatible, and low-cost pH stimulus-responsive polymer. It is a long-chained molecule extracted from the exoskeletons of crustaceans with pKa of ~6.5 [11]. Chitosan is hydrophilic and positively charged in acidic to neutral solution and readily binds to negatively charged surfaces, shifting to hydrophobic at pH values above its pKa [12]. This property enables the electrodeposition of chitosan from an aqueous bulk solution onto a conductive surface in response to cathodic signals requiring no crosslinking agents in a relatively quick process at room temperature [10,13]. It has also been shown that chitosan exerts multifunctional forces on bacteria such as hydrophobic, hydrogen bond, and electrostatic interactions that are complementary and can reinforce each other [14]. This property combined with the pH stimulus–response is thus shown to be beneficial for sensing bacteria; this study has demonstrated that the actuation of chitosan (pH stimulus) [15] and PNIPAAm (poly(N-isopropylacrylamide, a temperature-responsive polymer) [16] brushes can improve bacteria detection in complex aqueous media by increasing the probability of target–receptor interaction as a result of microscale structural changes. These previous works demonstrated that actuation of brushes with bacteria capture while brushes were in the extended state followed by brush collapse improve sensing performance (i.e., signal to noise ratio) [16]. 

A wide variety of sensor coating materials have been used in aptasensing. Attachment of aptamers to the sensor surface involves functionalizing the oligomer at the 5′ end with a tag (e.g., biotinylation, thiolation, etc.). Thiolation (conjugation of a thiol group) is one of the most versatile tagging methods and is highly useful in electrochemical sensing due to the coupling with noble metal(s), such as gold and platinum, on the sensor surface. Previous studies have co-electrodeposited chitosan with metal ions [17,18,19]. However, no work has been done on simultaneous electrodeposition of chitosan and platinum to fabricate stimuli-responsive nanostructures, nor its use to detect bacteria in food products. This work focuses on applying pulSED to a new one-step simultaneous electrodeposition of nanoplatinum and chitosan (CHI/Pt) to fabricate a biomimetic nanostructure that actuates under pH changes. The study shows the development of a rapid, label-free impedimetric aptasensor for real-time detection of *Listeria monocytogenes* in chicken broth, based on stimulus response of CHI/Pt brushes through a combination of electrostatic interactions and aptamer–cell binding. Additionally, we demonstrate that actuation of CHI/Pt brushes results in improved *Listeria* spp. capture and controllable electrochemical transduction based on an external stimulus (i.e., pH change) in the presence of interferents from food samples. This effect is likely due to polymer entrapment after initial aptamer binding to target cells.

## 2. Materials and Methods

### 2.1. Materials and Reagents

Chloroplatinic acid 8 wt.%, chitosan (low molecular weight, 75–85% deacetylated 20–300 cP), hydroquinone, hexaamineruthenium(III) chloride, potassium phosphate monobasic, and sodium phosphate dibasic were purchased from Sigma-Aldrich Co. (St. Louis, MO, USA). Polycrystalline diamond suspensions (1 µm and 3 µm) and alumina slurry (0.05 µm) were obtained from Buehler (Lake Bluff, IL, USA). Lead acetate (30% *w*/*v*) was purchased from Fisher Scientific (Pittsburgh, PA, USA). Potassium ferrocyanide trihydrate was purchased from Ward’s Science (Rochester, NY, USA). Potassium ferricyanide and cysteine hydrochloride monohydrate were obtained from J.T. Baker (Phillipsburg, NJ, USA). Platinum/iridium working electrodes (Pt/Ir, BASi MF-2013, 1.6 mm diameter, 7.5 cm length), reference electrode (Ag/AgCl) and Pt auxiliary electrode were purchased from BASinc. (West Lafayette, IN, USA).

Sulfo-SMCC (sulfosuccinimidyl 4-(N-maleimidomethyl)cyclohexane-1-carboxylate) and 1-ethyl-3-(3-dimethylaminopropyl)carbodiimide HCl (EDC) were obtained from Thermo Fisher Scientific (Waltham, MA, USA). N-Hydroxysuccinimide (NHS), 2-(morpholino)ethanesulfonic acid (MES) buffer, Ellman’s reagent (5,5′-dithiobis-(2-nitrobenzoic acid)), and platinum wire (99.95% Pt, 1.5 mm dia.) were obtained from Alfa Aesar (Ward Hill, MA). DiaEasy™ dialysis tubing (3.5 kDa MWCO) was purchased from Biovision (Milpitas, CA, USA).

*L. monocytogenes* aptamer developed by Ohk et al. [20] (47 DNA bases, K_D_ = 10^3^ CFU/mL, Mw 15,008 g/mol) terminated at the 5′ end with a thiol group was purchased from Gene Link (Hawthorne, NY, USA). Commercially sterilized chicken broth was purchased from a local grocery store. Tryptose phosphate broth (TPB) was acquired from HiMedia (Mumbai, India). Tryptic soy broth (TSB), tryptic soy agar (TSA), yeast extract, and buffered peptone water (BPW) were purchased from Becton, Dickson, and Company (Sparks, MD, USA). Potassium nitrate (KNO_3_) was purchased from British Drug Houses(BDH) (Mississauga, ON, Canada). Tris EDTA (TE) buffer pH 7.4 was purchased from Quality Biological (Gaithersburg, MD, USA). Sodium chloride (NaCl) and potassium chloride (KCl) were acquired from EMD Millipore Corporation (Burlington, MA, USA).

### 2.2. Bacteria Cultures

*Listeria monocytogenes* (ATCC 15313) and *Staphylococcus aureus* (ATCC 25923) were resuscitated from the frozen culture in TPB and TSB, respectively, using two identical consecutive transfers incubated at 35 °C for 24 h under aerobic conditions. Total aerobic plate counts were measured in triplicate by serially diluting samples of the bacteria in BPW and plating on TSA and TSAYE for *S. aureus* and *L. monocytogenes*, respectively. Bacteria were maintained on tryptic soy agar (TSA) and TSA with 0.6 wt.% yeast extract (TSAYE) slants for *S. aureus* and *L. monocytogenes*, respectively, and stored at 4 °C for no more than 3 months. *L. monocytogenes* and *S. aureus* are pathogenic microorganisms and must be handled using biosafety level 2 standards established by the National Institute of Health.

### 2.3. Nanomaterial Deposition 

**Figure 1:**
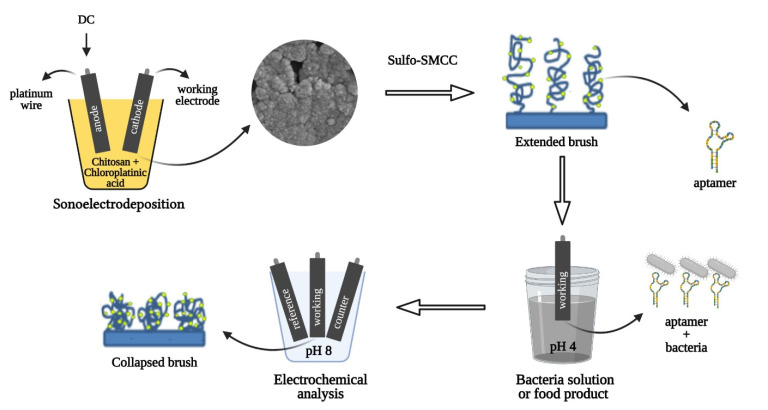
Fabrication, biofunctionalization, and sensing scheme of the platinum-decorated (CHI/Pt) aptasensor. CHI/Pt brushes were formed on the working electrode surface by a one-step sonoelectrodeposition method. Biofunctionalization with aptamers that target the protein Internalin A on *Listeria monocytogenes* membrane occurred via bonding between thiolated aptamer and nanoplatinum. Brush actuation for enhanced bacteria capture was facilitated by pH changes before and after electrochemical analysis.

The pulsed sonoelectrodeposition method (alternating applied potential and sonication in cycles of 1 s each) [9] was applied for the simultaneous deposition of chitosan and platinum onto the surface of Pt/Ir electrodes. For the deposition (see Figure 1), a platinum wire (positive or anode) and the working electrode (negative or cathode) were connected to the power supply and submerged in the deposition solution composed of 0.72 wt.% chloroplatinic acid, 0.001 wt.% lead acetate, and chitosan (nonmodified or thiolated) at different concentrations in distilled water. (See Supplementary Materials for the thiolated chitosan synthesis (CHI–thiomer)). Different deposition parameters and concentrations of chitosan were evaluated to determine the optimal deposition conditions. For the thiol-modified chitosan and platinum co-deposition (CHI–thiomer/Pt), it was used 0.05, 0.15, and 0.25 wt.% of chitosan suspended on the chloroplatinic acid solution; 2, 6, and 10 V of applied potential for 40, 60, and 80 cycles (deposition times of 80, 120, and 160 s, respectively). Based on the results of the CHI–thiomer/Pt, only 0.05 and 0.15 wt.% of chitosan, 6 and 10 V applied potential for 60 and 80 cycles were used for the simultaneous deposition of nonmodified chitosan and platinum (CHI/Pt).

### 2.4. Material Characterization 

Morphological characterization of the optimized depositions was conducted by scanning electron microscope (SEM) using a Quanta 600 FEG from FEI (Hillsboro, OR, USA). Electrodes were coated with a 5 nm thick layer of platinum using a Cressington sputter coater 208 HR (Watford, UK) and allowed to ventilate for 30 min prior to SEM imaging. Images were taken with an operating voltage of 10 kV at 10,000 and 30,100 times magnification for CHI/Pt brush deposition; and 5000 and 10,000 times for thiomer brushes.

X-ray photoelectron spectroscopy (XPS), also known as electron spectroscopy for chemical analysis (ESCA), was used to analyze the surface chemistry of the selected optimized conditions for the deposition of CHI/Pt and thiomer brushes. XPS was performed in an Omicron ESCA+ (Scienta Omicron, Uppsala, Sweden) equipped with a Mg/Al dual X-ray gun and CN10 electron gun.

### 2.5. Electrochemical Analysis

Cyclic voltammetry (CV) and electrochemical impedance spectroscopy (EIS) methods, as described by Hills et al. [15] and Giacobassi et al. [16], were used as noted. A three-electrode system with a platinum auxiliary electrode and an Ag/AgCl reference electrode at room temperature was used with a CHI 600E potentiostat analyzer. Cottrell plots were used to calculate the electroactive surface area (ESA) via Randles-Sevcik theory based on CV. ESA was used to evaluate the best deposition parameters for each polymer (CHI and thiolated-CHI), pH stimulus (4 and 8), and attachment of different concentrations of aptamers (500 to 1500 nM). CV analysis was conducted in 4 mM [Fe(CN)_6_]^3−^ with 1 mM KNO_3_ using a 10 s quiet time, a 650 mV switching potential, and scan rates of 50, 100, 150, and 200 mV/s [9]. 

Where noted, various redox probes were used to study changes in electrochemical behavior that are correlated with electrostatic interactions. Hydroquinone was used as a model neutral probe, hexaamineruthenium(III) chloride was used as a model positive probe, and potassium ferrocyanide trihydrate was used as a model negative probe. The actuation tests were conducted with 4 mM redox probes—in 1 mM KNO_3_ solution, and the pH was adjusted to pH 8 or pH 4, using a 1 M HCl or NaOH solution. The pH of the redox solutions was monitored during the tests to ensure the reported pH did not change by more than 0.5 pH units.

EIS was used to measure impedance differences caused by bacteria presence (ranging from 10^0^ to 10^6^ CFU/mL). (For details of the functionalization procedure of CHI/Pt and CHI–thiomer/Pt brushes with aptamers, see Supplementary Materials). The same electrode was used for the entire experiment to build the calibration curve, keeping the electrode in the solution with bacteria at pH 4 for 15 min and reading the EIS in a pH 8 solution without bacteria. At least three independent experiments were carried out to generate data for the calibration curve. Non-Faradaic EIS was performed in phosphate buffer saline (PBS) or chicken broth with a frequency range of 1–100,000 Hz, AC amplitude of 100 mV, and initial DC potential of 0 V. Bode plots (total impedance versus frequency) were used to perform the cutoff frequency analysis and to derive calibration curves. These calibration curves consisted of the change in impedance (Δ$Z=Z_{bacteria}-Z_{no\;bacteria}$) (Ohm) vs. the concentration of cells (log CFU mL^−1^) at fixed cutoff frequencies as noted. The limit of detection (LOD) was calculated using the 3σ method, and the analytical sensitivity was determined by the slope of the linear portion of the calibration curve (R^2^ > 0.98) at a fixed frequency [16]. Analysis with the presence of interferents (food components and other bacteria) was assessed to determine the selectivity for target bacteria. 

### 2.6. Statistical Analysis

All experiments were performed at least in triplicate and analyzed using one-way analysis of variance and ANOVA to test for significance. Statistical significance was expressed at the *p* < 0.05 level; significantly different means were classified using a Tukey test.

## 3. Results and Discussion

### 3.1. Nanobrush Material Characterization

**Figure 2:**
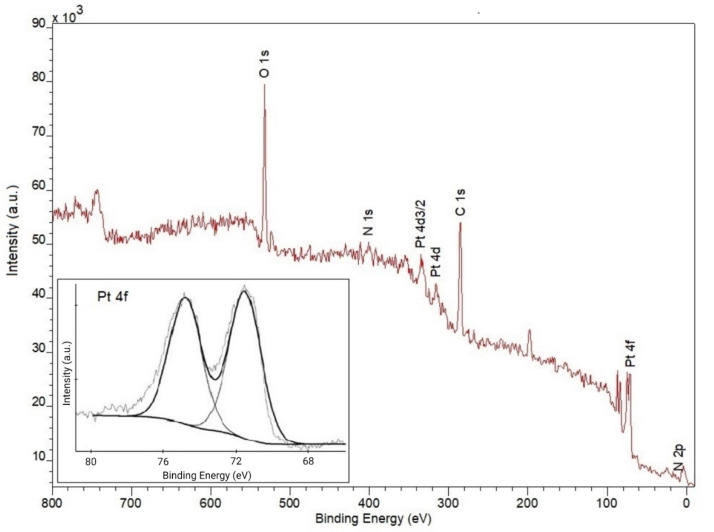
X-ray photoelectron spectroscopy (XPS) survey spectrum of the CHI/Pt brush deposition, demonstrating that both chitosan and platinum were successfully deposited with the presence of C 1s, O 1s, N 1s, and Pt 4f and Pt 4d peaks. Inset shows the Pt 4f spectrum.

XPS spectrum of the CHI/Pt brushes (Figure 2) shows the effective co-deposition of both chitosan and platinum-based on the presence of C 1s, O 1s, N 1s, and Pt 4f and Pt 4d peaks. C 1s peaks between 285 eV and 286.5 eV are assigned to CH_2_ alkyl chain backbone, C–NH_2_, and C–O species [17,21]. The N 1s binding energy between 399 eV and 401 eV are consistent with that of C-N species of the amine group at 399 eV and protonated amine group at 401 eV [10,21]. Hwang et al. [17] assigned a peak at 399.3 eV to the amino group involved in the hydrogen bond and a peak at 401.7 eV to the chelation between the amino group and bismuth metal. From the platinum co-deposition, the Pt 4f spectrum shows a characteristic doublet with binding energies of 71.5 eV and 74.8 eV (Figure 2 inset). Some authors associate the binding energy around 71–71.5 eV with metallic Pt^0^ [22,23], while others described a doublet as typical for the 7/2 and 5/2 spin states for pure Pt nanoparticle in the zero-valent state at lower binding energies (70 eV and 73.3 eV, respectively) [24,25]. A doublet at 71.1 eV and 74.4 eV is also observed for control studies using only platinum deposited onto the surface from chloroplatinic acid solution (data not shown). See Figure S4 in the Supplementary Materials for the XPS spectrum of the CHI–thiomer/Pt brush.

**Figure 3:**
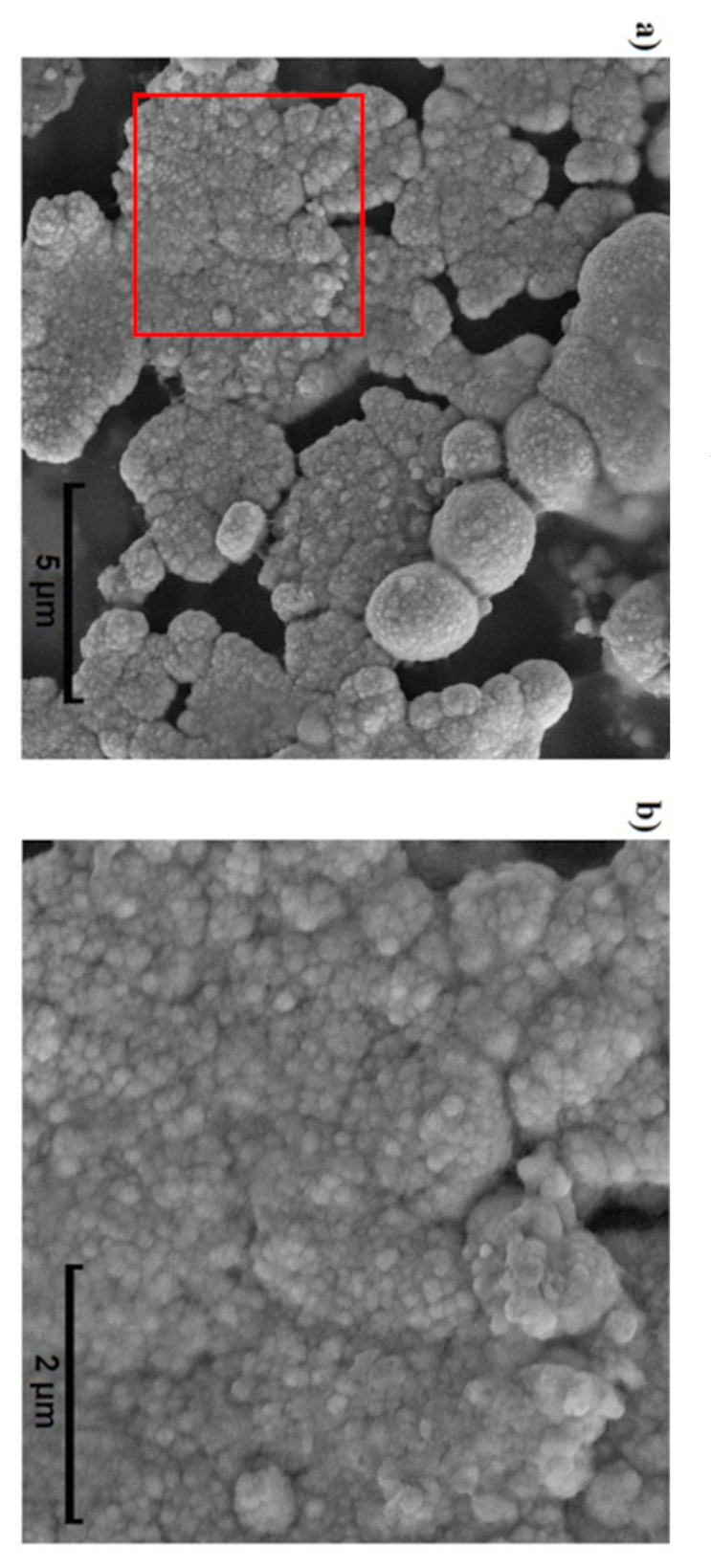
Morphological characterization by scanning electron microscopy (SEM) images of CHI/Pt brushes at 10 kV and (**a**) 10,000, and (**b**) 30,100 times magnification (red square in (**a**)) show a heterogeneous surface with irregular distribution of CHI/Pt brushes.

A polymer brush consists of end-tethered (grafted, anchored) polymer chains stretched away from the substrate, and its shape depends on the deposition method, substrate, and solvent, among others [26]. Liu et al. [27] studied Pt electrodeposition showing that pulse duration affects surface morphology, and for pulses of 1 s, as in the present work, the electrodeposition process is controlled by the diffusion process since the consumed Pt ions cannot be compensated at this pulse rate. Consequently, the Pt nuclei tend to grow into a 2D planer structure, which results in a relatively large electroactive surface area (ESA) (discussed in Section 3.2). In the present work, the simultaneous deposition of CHI/Pt brushes resulted in a heterogeneous distribution of nanobrushes and nonuniform formation of brushes with sizes between 80 and 250 nm on the electrode surface that resembled spheroids composed of smaller spheroids (Figure 3). Overall, the microstructures were less uniform than what was observed by Liu et al. [27], and the morphology in this work was smoother, which is likely due to the chitosan co-deposition. Hills et al. [15] showed that electrodeposition of chitosan over a reduced graphene/platinum layer presented a more brush-like morphology with visible longitudinal shafts and larger terminal nodes (200–300 nm) than the work here. Giacobassi et al. [16] reported the size of PNIPAAm nanobrush terminal nodes between 220 and 1300 nm with a similar spheroid structure as the CHI/Pt brushes observed in this study, although more uniformly distributed. Additional images for the CHI/Pt brush deposition are available in the Supplementary Materials (Figure S1), as well as images of the CHI-thiomer/Pt brush deposition (Figure S5).

### 3.2. Electrochemical Characterization 

**Figure 4:**
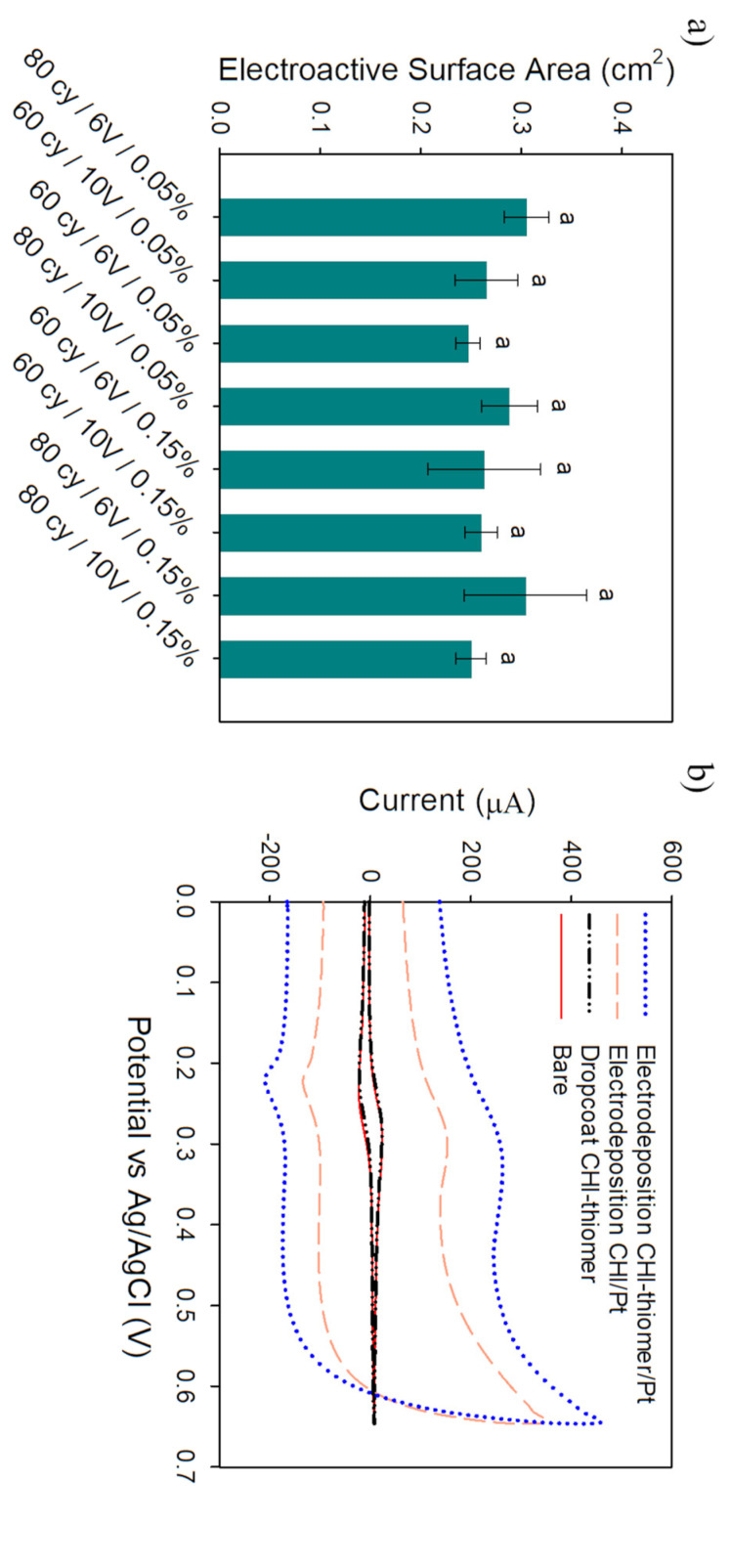
Electrochemical characterization of electrodes using 4 mM K_4_FeCN_6_ as a redox probe. (**a**) Average electroactive surface area (ESA) for various electrode modifications with a different number of cycles (cy), voltages (V), and chitosan concentration (wt.%). (**b**) Representative CV curves at 100 mV/s scan rate of bare Pt/Ir, drop-coated CHI-thiomer, and best conditions for CHI/Pt (80 cycles/6 V/0.05% CHI) and CHI-thiomer/Pt (60 cycles/6 V/0.15% CHI-thiomer) brush depositions. All data represent the average of three replicates, and error bars represent the standard deviation of the arithmetic mean; letters denote significantly different means (*p* < 0.05).

Cyclic voltammograms in 4 mM K_4_FeCN_6_ and 1 M KNO_3_ (pH = 7.1, T = 25 °C) were obtained in order to determine the optimal brush deposition conditions. Figure 4a shows the average electroactive surface area (ESA) for electrodes treated with CHI/Pt brushes. Figure 4b shows representative cyclic voltammetry (CV) curves of the best conditions for CHI/Pt and CHI-thiomer/Pt brushes (more results available in Figure S6 in the Supplementary Materials) compared to bare Pt/Ir electrode and drop coating of CHI-thiomer without Pt co-deposition at the bare electrode’s surface. The thiol groups on the CHI-thiomer did not promote enough attachment onto the electrode’s surface by drop coating, i.e., no significant change (*p* < 0.05) in current peak value (Figure 3b) and, consequently, on ESA value. The experiments with CHI-thiomer/Pt brush were the first ones to be performed, and results demonstrated reduced (*p* < 0.05) ESA values at a lower voltage (2 V) and number of cycles (40 cycles) as well as higher CHI-thiomer concentration (0.25 wt.%) (see Figure S6 in Supplementary Materials), consequently these conditions were eliminated for the experiments with CHI/Pt brushes. 

Chitosan is positively charged when soluble (below its pKa) and can be electrodeposited via a cathodic neutralization mechanism [28]. Previous studies demonstrated that chitosan-based hydrogels could be deposited onto a cathode surface as the applied voltage promotes proton-consuming hydrogen evolution reaction at the cathode surface [13,19,29,30]. This leads to a gradual increase of the pH near the cathode surface, and if this localized pH exceeds 6.3, then chitosan becomes insoluble and deposits at the cathode surface [29,31]. Nevertheless, as the current density increases, the region of high pH is expected to extend further from the cathode surface into the bulk solution [29]. It has been reported that the thickness of the deposited chitosan film was dependent upon the applied voltage, the chitosan concentration, and the deposition time [30,31]. For instance, Luo et al. [31] found that longer deposition time (10 min) resulted in tight attachment of thick films and longer response time of the resulting biosensors, while shorter deposition time (1 to 3 min) resulted in thin and unstable chitosan film and shorter response time of the resulting biosensors. Hills et al. [15] stated that electropolymerization of chitosan for 2 min resulted in nanobrushes with no appreciable stimulus response, while polymerization times longer than 6 min resulted in nanobrushes that did not adhere well to reduced graphene oxide/platinum electrodes. In the present work, on preliminary tests, deposition times longer than 160 s (80 cycles) resulted in deposition beyond the working diameter of the electrode. Taguchi et al. [9] experienced a similar result with pure Pt electrodeposition over 180 s and stated that this “overgrowth” might not be stable and flake off when immersed in the solution. Cheng et al. [13] also reported a lateral expanding thickness of electrodeposited chitosan exceeding the electrode edge with increasing deposition times and current densities. As shown in Figure 4a, despite all the different deposition conditions tested, there were no significant (*p* > 0.05) differences among the ESA values obtained. Consequently, 6 V was selected for further experiments of both CHI/Pt and CHI-thiomer/Pt brushes since overgrowth was often observed with 10 V co-deposition. The simultaneous deposition of Pt nanoparticles with the chitosan seems to have a more pronounced effect on overgrowth occurrence. 

For the selected optimum condition of 80 cycles/6 V/0.05% CHI, the electrodeposition of CHI/Pt brushes onto electrodes increased (*p* < 0.05) the average ESA by 11 times (0.31 ± 0.02 cm^2^) from the bare Pt/Ir electrode (0.028 ± 0.003 cm^2^). This ESA value is similar to previous work by Taguchi et al. [9] with an ESA value of 0.3 cm^2^ when depositing platinum nanoparticles using the same pulSED technique used in the present work with higher voltage and deposition time (10 V and 90 cycles). Hills et al. [15] presented ESA about six times lower (0.048 ± 0.017 cm^2^) than the present work with chitosan being electrodeposited onto a reduced graphene oxide/nano-platinum coated electrode. The improved ESA results obtained here with the simultaneous electrodeposition of chitosan and platinum might be attributed to the properties of chitosan related to the formation of stable chelates with many transition metal ions due to the presence of hydroxyl and amino groups, which provide enhanced affinity to metal ions and improved detection sensitivity [17,32].

Some reports on the use of metals and chitosan together to produce sensors are available in the literature, most of them with two or more steps for the deposition, either layer by layer deposition [15] or by forming a metal–chitosan complex prior to deposition [18]. Hwang et al. [17] performed a co-electrodeposition of bismuth and chitosan to detect heavy metals in wastewater. Reduced graphene oxide and chitosan were co-electrodeposited by chronoamperometry [33]. The mechanism of co-electrodeposition has been associated with the ability of chitosan to coordinate with metal ions due to the presence of amino groups [34]. However, to the best of our knowledge, simultaneous electrodeposition of chitosan and platinum to fabricate stimuli-responsive biomimetic nanostructures has not been described before, nor its use to detect bacteria in food products. 

### 3.3. Actuation of Nanobrushes

**Figure 5:**
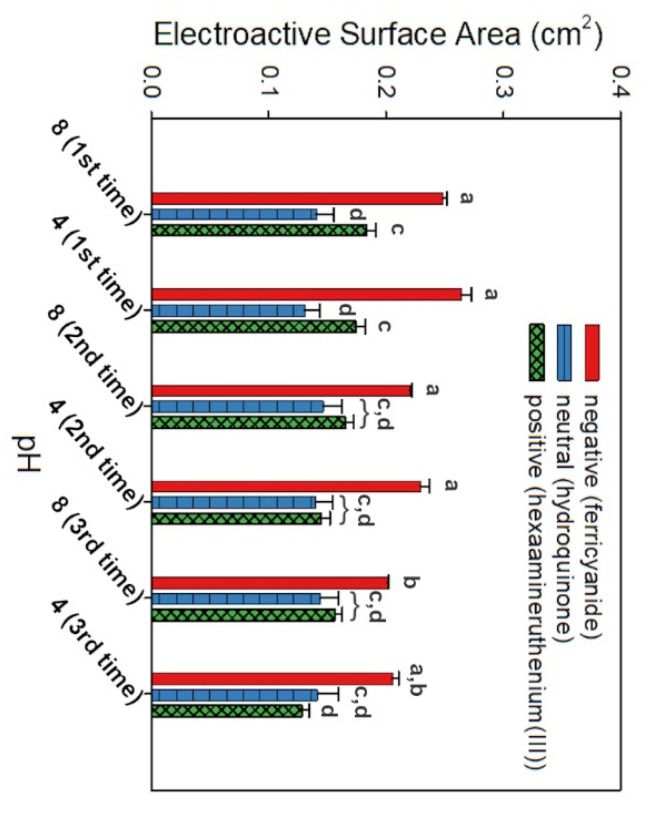
Electrostatic interactions during chitosan actuation for various redox probes: a negatively charged probe (KFeCN_6_^3−^), a neutral probe (C_6_H_4_(OH)_2_), and a positively charged probe (Ru(NH_3_)_6_^3+^. The average electroactive surface area (ESA) is shown for each redox probe under repeated actuation at pH 4 and pH 8 (*n* = 3), indicating some degree of hysteresis. Error bars represent the standard error of the arithmetic mean; letters denote significantly different means (*p* < 0.05).

Chitosan is a polycationic polymer with pKa around 6.5 [35]. Below pKa, chitosan’s amines are protonated, the polymer has a high charge density, and electrostatic repulsions between monomeric units tend to stiffen the chain leading to an extended conformation [13,32]. At higher pH, the amines become deprotonated and ionic repulsions are reduced, thus allowing the individual chains to collapse [13]. To characterize the electrostatic interactions during polymer actuation, CV was performed with three different redox probes at various pH, including a negatively charged probe (KFeCN_6_^3−^), a neutral probe (C_6_H_4_(OH)_2_), and a positively charged probe Ru(NH_3_)_6_^3+^. Figure 5 shows the average ESA values for replicate sensors at pH 4 or 8 during three repetitive cycles. At lower pH, electron transport increases with the negative probe (KFeCN_6_^3−^) due to electrostatic interactions with positively charged chitosan; and with the positive probe (Ru(NH_3_)_6_^3+^), the opposite occurs due to charge repulsion/steric hindrance. Above the pKa (pH 8), the trend in CV data is reversed, with a reduction of peak current on the negative probe, while with the positive probe, the electron transfer is favored increasing the ESA. Due to the lack of charge of the neutral probe, the influence of pH is almost negligible. Similar results were observed previously by Hills et al. [15] with nonmodified chitosan electrodeposited onto a reduced graphene oxide-nanoplatinum-coated electrode, which demonstrates that simultaneous deposition of chitosan with platinum studied here does not affect chitosan’s stimulus response to pH changes and actuation properties. Some degree of hysteresis can be observed in Figure 5 after repeated actuation of the CHI/Pt brush, as the ESA gradually decreases after each repetition. This behavior was also noticed by Giacobassi et al. [16] during the actuation of PNIPAAm nanobrushes with temperature stimuli. Actuation tests were also performed with the CHI-thiomer/Pt brush electrodes to test if the modification of chitosan had any effect on its pH stimuli actuation property (see Figure S6 in Supplementary Materials). 

Hills et al. [15] and Giacobassi et al. [16] demonstrated that the actuation of stimuli-responsive polymers in stagnant media improves bacteria capture (relative to no actuation). The authors reported improved sensing performance when cell capture was in the extended conformation during capture (EX/cap), followed by the collapse of the nanobrush during the measurement (COL/meas). This could be due to (1) the increased probability of aptamer–cell interaction in the extended phase, and (2) electrostatic attraction between the CHI and *L. monocytogenes* below pH 6, as CHI is positively charged and under most conditions, *L. monocytogenes* membrane is highly negatively charged [15,36]. Based on the actuation results obtained in this study that corroborate with the results obtained by Hills et al. [15] and Giacobassi et al. [16], the previously established actuation protocol (i.e., EX/cap followed by COL/meas) was used for the sensing experiments with bacteria described in the next section.

### 3.4. Bacteria Sensing

**Figure 6:**
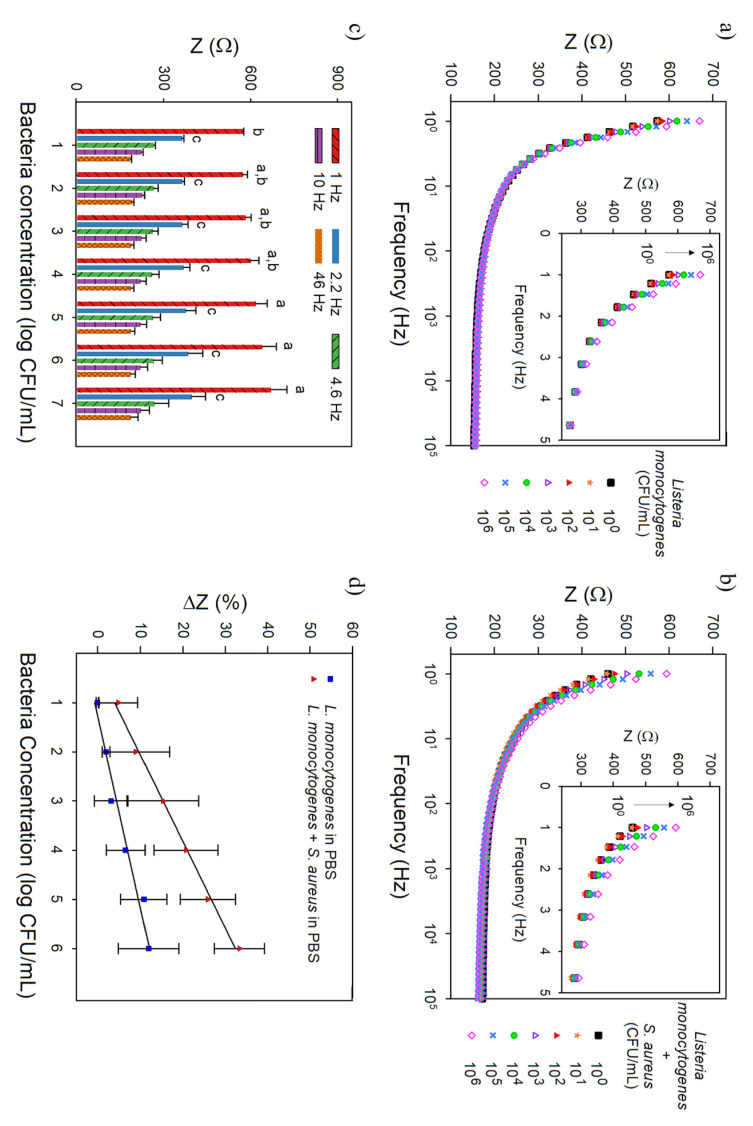
Representative Bode plots over the frequency range of 1–100,000 Hz (inset show exploded view over the frequency range from 1–5 Hz) for the CHI/Pt brush sensor functionalized with 1000 nM aptamer exposed to (**a**) *L. monocytogenes* in PBS and (**b**) *L. monocytogenes* + *S. aureus* in PBS. (**c**) The average impedance at different cutoff frequencies with *L. monocytogenes* in PBS. There is no significant difference among frequencies 4.6, 10, and 46 Hz, but the other cutoff frequencies (1.0 Hz, 2,2 Hz) are significantly higher. (**d**) Calibration curves (impedance change at 1 Hz vs. log bacteria concentration) for (**a**,**b**). All data represent the average of three repetitions. Error bars represent the standard deviation; letters in the plot (**c**) denote significantly different means (*p* < 0.05).

**Figure 7:**
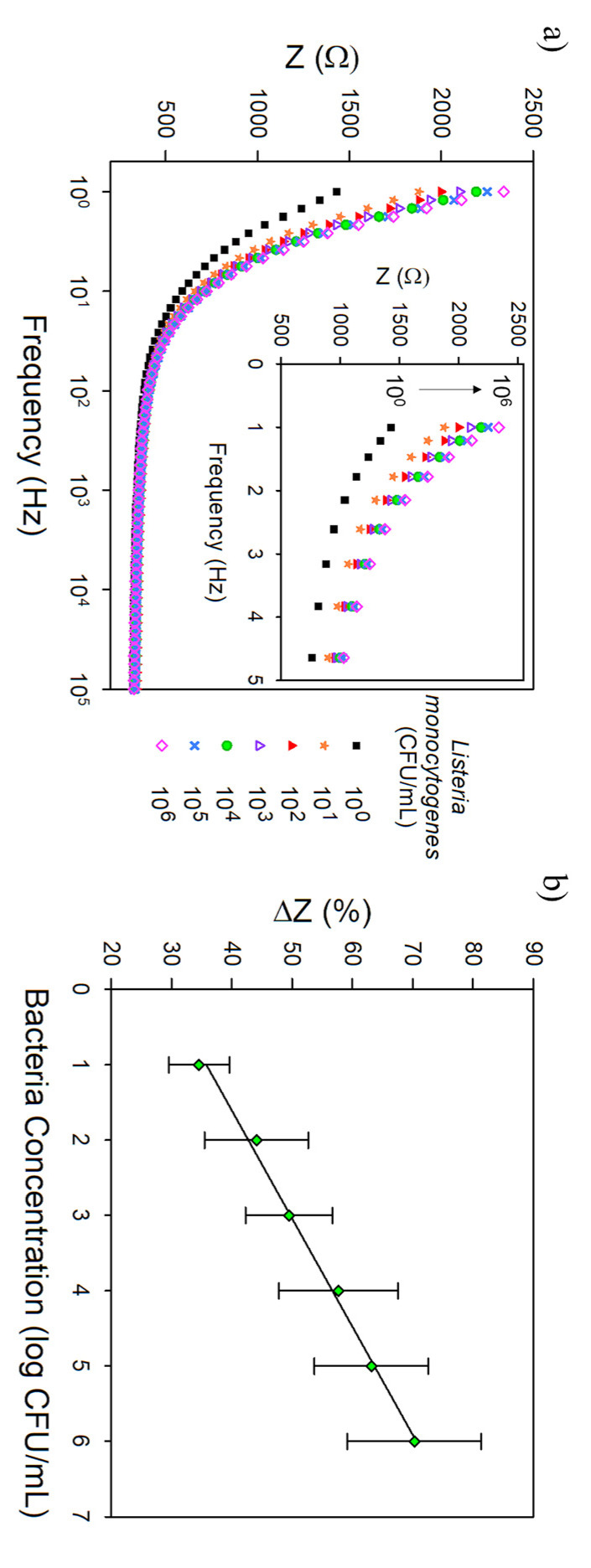
(**a**) Representative Bode plot over the frequency range of 1–100,000 Hz (inset show exploded view over the frequency range from 1–5 Hz) and (**b**) calibration curve (impedance change at 1 Hz vs. log bacteria concentration) for the CHI/Pt brush sensor functionalized with 1000 nM aptamer exposed to *L. monocytogenes* in chicken broth. All data represent the average of three repetitions. Error bars represent the standard deviation.

Based on the principle that binding of target bacteria to the aptamer decreases the electron transfer at the electrode measured as an increase of impedance, the EIS analysis was performed to determine impedance differences caused by bacteria presence. All tests used the actuation protocol of capture in the extended state at pH 4 and sensing in the collapsed state at pH 8 [15,16] and included 15 min for bacteria capture and 2 min for the EIS measurement. The CHI/Pt brush electrodes functionalized with an aptamer concentration of 1000 nM were tested with increasing concentration of *L. monocytogenes* (ranging from 100 to 10^6^ CFU/mL). See Supplementary Materials for details on the nanobrush functionalization procedure and results (Figure S7). Bode plots (Figure 6 and Figure 7) are shown over a frequency range of 1 Hz to 100 kHz; insets are a zoom-in view of the lower frequency range (1–5 Hz).

Bacteria detection was first assessed in PBS at room temperature. As observed on the Bode plots (Figure 6a,b), at higher frequencies (above 10 Hz), the impedance values overlapped, while at lower frequencies, the impedance values consistently increased with the increase in bacteria concentration. For the frequency cutoff (CF) analysis (Figure 6c), the maximum impedance signal was obtained at a CF of 1 Hz, which was used to determine the calibration curves and limit of detection (LOD, 3 sigma; 99.5% confidence interval). This analysis considered frequencies from 1 to 50 Hz, which falls within the alpha frequency dispersion region in biological tissues [37].

Figure 6d shows that impedance increased linearly for the CHI/Pt-aptamer nanohybrid electrode when calibrated in PBS with *L. monocytogenes* and in the presence of equal background concentration of *S. aureus* (interferent) with a range of detection of 10^1^ to 10^6^ CFU/mL. *S. aureus* was the bacteria chosen for selectivity testing due to its similarity to *Listeria*, with both being Gram-positive and both being known foodborne pathogens. The LOD of the CHI/Pt-aptamer brushes when only *Listeria* was present in PBS was 2.5 ± 0.3 CFU/mL, and its sensitivity was 21.1 ± 4.4 Ω/log(CFU/mL). The addition of *S. aureus* in the testing solution did not show significant interference (*p* > 0.05) on the CHI/Pt-aptamer brushes performance, with LOD of 2.6 ± 1.6 CFU/mL and sensitivity of 27.8 ± 3.5 Ω/log(CFU/mL), which indicates no crossreaction between *S. aureus* and the aptamer, and consequently high aptamer selectivity to *L. monocytogenes*. 

The results of the CHI/Pt brush not functionalized with the aptamer (plots presented in Figure S2 in Supplementary Materials), in PBS with only *L. monocytogenes*, the LOD, and sensitivity (3.1 ± 0.0 CFU/mL and 26.7 ± 5.5 Ω/log(CFU/mL), respectively, are similar (*p* > 0.05) to CHI/Pt–aptamer brush. The inclusion of *S. aureus* significantly increased the LOD (11.0 ± 0.4 CFU/mL), reduced the sensitivity (11.3 ± 1.2 Ω/log(CFU/mL)), and the detection range to 10^1^ to 10^5^ CFU/mL for CHI/PT brush without aptamer functionalization. Abdelhamid and Wu [14] showed that the interactions between chitosan and bacteria are entropically driven, and the binding processes are spontaneous. The authors determined that chitosan has multifunctional forces on bacteria (hydrophobic, hydrogen bond, and electrostatic interactions), which are complementary and can reinforce each other. This can explain the higher LOD when the CHI/Pt brushes without aptamer functionalization are tested with both *L. monocytogenes* and *S. aureus* in PBS as both bacteria are prone to bind to chitosan. Additionally, these results demonstrate that the actuation protocol plays an important role in bacteria capture and can be used as the first step in food safety monitoring. These results also reinforce that the aptamer is highly selective to *L. monocytogenes* improving the overall CHI/Pt-aptamer brush sensing performance. 

Previous studies have used the high electrostatic affinity of positively charged chitosan toward negatively charged bacterial cell membranes to detect bacteria. Le et al. [38] developed a colorimetric method using the peroxidase-like activity of chitosan-coated iron oxide magnetic nanoparticles that allowed the rapid detection of bacterial cells (*Escherichia coli* and *Staphylococcus aureus*) down to 10^4^ CFU/mL by the naked eye and 10^2^ CFU/mL by spectrophotometry within 10 min. Abdelhamid and Wu [39] also reported detection of 10^2^ CFU/mL *Pseudomonas aeruginosa* and *S. aureus* in blood samples using a graphene magnetic nanosheet decorated with chitosan biosensor and fluorescence spectroscopy as a transduction method.

Figure 7 shows the analysis results in chicken broth to evaluate the CHI/Pt-aptamer brush sensing performance in real food. Chicken broth was chosen as an example of complex media that presents carbohydrates and proteins, among other components, that could interact with the biosensor through nonspecific adsorption resulting in a false-positive signal. The LOD remained similar (3.3 ± 0.9 CFU/mL, *p* > 0.05) and the sensitivity significantly increased (89.3 ± 0.4 Ω/log(CFU/mL)) when testing in chicken broth compared with sensing in PBS. When the CHI/Pt brushes without aptamer were tested in the same conditions (Figure S3 and Table S1 in the Supplementary Materials), the LOD was significantly higher (22.0 ± 1.3 CFU/mL) and the sensitivity lower (43.1 ± 13.3 Ω/log(CFU/mL)). One more time demonstrating that the aptamer efficiency to selectively bind to *L. monocytogenes* even in the complexity of a food matrix and the ability to capture bacteria applying the actuation protocol to the CHI/Pt brushes.

The CHI–thiomer/Pt-aptamer nanohybrid electrodes provided very inconsistent results when tested with bacteria (Figure S8 in Supplementary Materials). The electrostatic forces between the negative charges of the bacteria and polycationic sites of chitosan [14] can explain the better performance of the nonmodified chitosan than the CHI-thiomer, as some of the cationic sites of chitosan were unavailable after the modification.

Some reports in the literature used chitosan combined with other materials to detect heavy metals, food colorants, cancer biomarkers, among others [17,40]. Table 1 contains a compilation of the application of chitosan and aptamers to detect different bacteria in various food samples or buffers. The detection limit and detection time for our actuating CHI/Pt brush sensor were less than or equal to the LODs and response times reported in the literature, except for Zelada-Guillén et al. [41], who reported an aptasensor for *E. coli* CECT 675 in milk with a LOD of 6 CFU/mL and detection time ranging from 2 to 20 min. For *Listeria* spp. detection, the LOD of CHI/Pt brush sensor was lower than all biosensors shown in Table 1 with similar detection times. Radhakrishnan et al. [42] achieved a LOD of 4 CFU/mL for *L. monocytogenes* in filtered tomato extract with a gold electrode-based immunosensor and EIS acquisition time of 2.8 min; however, with an electrode area (0.19 cm^2^) ten times larger than our electrode. Wang et al. [43] developed a TiO_2_ nanowire bundle based immunosensor functionalized with anti-*Listeria* antibodies that can detect as low as 10^2^ CFU/mL and up to 10^7^ CFU/mL of *L. monocytogenes* in 1 h without significant interference from other foodborne pathogens; however, no linear correlation was found. Guo et al. [44] proposed aptamer and antibody-based dual recognition units using aggregation-induced emission nanoparticles and magnetic nanoparticles and reported a detection range for *L. monocytogenes* of 10–10^6^ CFU/mL after a 90 min incubation time. Using the same aptamer as in the present work, Ohk et al. [20] presented a LOD of 10^3^ CFU/mL for a fiber-optic biosensor to detect *L. monocytogenes*. Sidhu et al. [45] reported a LOD of 5.39 CFU/mL of *L. monocytogenes* in PBS with the same detection time and the same aptamer as in this work using platinum interdigitated array microelectrodes. Hills et al. [15] studied an actuating nanobrush consisting of reduced graphene oxide/nano-platinum/chitosan functionalized with the same aptamer used in the present work and reported similar LOD values (3.0 and 9.1 CFU/mL in PBS and vegetable broth, respectively). One of the main advantages of the CHI/Pt brush studied here is the faster fabrication with only one-step deposition followed by functionalization, while Hills et al. [15] used a 3-step fabrication procedure before functionalization. The performance of the nanobrush biosensors developed here demonstrates that this device has the potential to be used in similar complex solutions, including brine water, aquaponics water, and other food samples that are suspended in water and diluted. A significant advantage of the biosensors in this work is the lack of bacteria purification or concentration steps, see, for example, Heo et al. [5] that required 4 h incubation prior to DNA extraction to achieve a detection limit of 10^0^ to 10^2^ CFU/g or mL, depending on the food, by real-time PCR.

## 4. Conclusions

There is an urgent need for a rapid, reliable, and cost-effective method for detecting foodborne pathogens in the food industry as public health and economic losses due to disease outbreaks and recalls of contaminated food are a real concern. A facile method for fabrication of chitosan–nanoplatinum brush sensing platform using a co-electrodeposition procedure was successfully demonstrated. The use of chitosan with nanoplatinum brush interfaces in combination with aptamers was shown to significantly enhance the capture of target *L. monocytogenes* bacteria and transduction of electrochemical signal as the acquisition method. The actuation protocol consisted of capturing bacteria at pH 4 when chitosan nanobrushes were extended and initiating test sequence at pH 8 when the brushes were collapsed, demonstrating the efficiency of these brushes in capturing the bacteria even in the absence of a biorecognition agent (i.e., *Listeria* aptamer). The results suggest they could be used as a first step for food safety monitoring. Expanded brushes exposed both biorecognition agents and chitosan amine groups to bacteria to easily bind, while collapsed brushes assisted in electrochemical signal transduction. The combination of aptamer binding and polymer entrapment is the likely mechanism for the improved sensing performance. 

Optimized conditions provided a sensitive, selective, and label-free sensor for detecting low concentrations of *L. monocytogenes*, even in the presence of interferents. The relatively high sensitivity of the developed sensor could be attributed to the combination of chitosan, which has a strong affinity towards bacteria due to the presence of an amine group (-NH_2_), and aptamers that are developed to bind specifically to a target membrane protein in *L. monocytogenes*. The LOD of CHI/Pt-aptamer brush sensor for *L. monocytogenes* in PBS was 2.5 CFU/mL (sensitivity of 21.1 ± 4.4 Ω/log(CFU/mL)) and, to simulate a real-world complex sample, chicken broth was used, which resulted in a LOD of 3.3 CFU/mL (sensitivity of 27.8 ± 3.5 Ω/log(CFU/mL)). Other sensors in literature may have comparable detection limits and sensitivities to those obtained in the present work; however, the developed sensor has several advantages: (1) preparation involves only one-step deposition, (2) no labeling necessary, (3) no bacteria concentration required (it only requires control of the pH on the sample and measurement solutions), and (4) rapid response time (17 min including sample exposure and testing). Additionally, the use of aptamers over antibodies regarding the production cost and shelf-life make CHI/Pt-aptamer brush sensor a potential alternative to current detection methods for testing food samples. Overall, the sensing platform developed in this study could be customized with different biorecognition agents for future electrochemical sensing applications.

## Figures and Tables

**Table 1:** Biosensor performance comparison to other devices in the literature using chitosan and aptamer for bacteria detection.

Biorecognition Agent and Platform	Bacteria	Detection Mode	Test Medium	Detection Range * (CFU/mL)	Response Time (min)	Reference
CHI/Pt + aptamer	*L. monocytogenes*	Impedimetric	PBS	2.5–10^6^	17	This work
CHI/Pt + aptamer	*L. monocytogenes*	Impedimetric	PBS + *S. aureus*	2.6–10^6^	17	This work
CHI/Pt + aptamer	*L. monocytogenes*	Impedimetric	Chicken broth	3.3–10^6^	17	This work
rGO-nPt + CHI + aptamer	*L. monocytogenes*	Impedimetric	Vegetable broth	9.1–10^2^	17	Hills et al. [15]
Pt-IDEs + aptamer	*L. monocytogenes*	Impedimetric	PBS	5.39–10^6^	17	Sidhu et al. [45]
GCE-NCs + aptamer	*P. aeruginosa*	Impedimetric	PBS	3–10^7^	30	Sarabaegi and Roushani [46]
rGO-CHI + aptamer	*Salmonella* Typhimurium	DPV	PBS	10–10^7^	Not reported	Dinshaw et al. [33]
AuNC-chitosan + aptamer	*S. aureus*	Colorimetric	PBS	10^2^–10^7^	>35	Xie et al. [47]
SWCNT + aptamer	*E. coli* *CECT 675*	Potentiometric	Milk Apple juice	6–10^4^ 26–10^4^	2–20	Zelada- Guillén et al. [41]
SWCNT + aptamer	*Salmonella Typhi*	Potentiometric	PBS	1–10^3^	1–30	Zelada- Guillén et al. [48]

* lowest value corresponds to the lower limit of detection (LOD). rGO: reduced graphene oxide; nPt: platinum nanoparticle; GCE: glassy carbon electrode; NC: nano-sized chitosan particles; AuNC: gold nanoclusters; DPV: differential pulse voltammetry; SWCNT: single-walled carbon nanotubes.

## Data Availability

The data presented in this study are available on request from the corresponding authors.

## Supplementary Materials

The following are available online at https://www.mdpi.com/article/10.3390/bios11120511/s1, Figure S1. SEM images of CHI/Pt brushes at 10 kV. (a) 10,100 and (b) 15,000 times magnification showing a heterogeneous surface with the nonuniform distribution of CHI/Pt brushes, featuring spheroid structures; Figure S2. Representative Bode plot over the frequency range of 1–100,000 Hz (inset shows enlarged view over the frequency range from 1–5 Hz) for the CHI/Pt brush sensor without functionalization with aptamer exposed to (a) *L. monocytogenes* in PBS, (b) *L. monocytogenes* and *S. aureus* in PBS, and c) calibration curves; Figure S3. Representative Bode plot over the frequency range of 1–100,000 Hz (inset show exploded view over the frequency range from 1–5 Hz) for the CHI/Pt brush sensor without functionalization with aptamer exposed to (a) *L. monocytogenes* in chicken broth and (b) calibration curve (impedance change at 1 Hz vs. log bacteria concentration). All data represent the average of three repetitions. Error bars represent the standard deviation; Table S1. Performance of the CHI/Pt brush biosensor without functionalization with aptamer when exposed to *Listeria monocytogenes* in different media; Figure S4. X-ray photoelectron spectroscopy (XPS) survey spectrum of the CHI/Pt brush deposition. Inset shows the Pt 4f spectrum indicating the presence of S 2p peak unbound Pt^0^ and S-bonded Pt; Electrochemical characterization of CHI-thiomer/Pt brushes; Figure S5. SEM images of CHI-thiomer/Pt brushes at 10 kV and (a) 5100 and (b) 10,100 times magnification indicate a nonhomogenous and dispersed brush distribution with varying brush sizes resembling a network of spherical particles with diameters between 100 and 1000 nm; Figure S6. Electrochemical characterization of CHI-thiomer/Pt brushes. (a) Average electroactive surface area (ESA) for various electrode modifications with a different number of cycles (cy), voltages (V), and CHI-thiomer concentration (wt.%) using 4 mM K_4_FeCN_6_ as a redox probe. (b) Electrostatic interactions during CHI-thiomer/Pt brush actuation for various redox probes: a negatively charged probe (KFeCN_6_^3−^), a neutral probe (C_6_H_4_(OH)_2_), and a positively charged probe (Ru(NH_3_)_6_^3+;^ Figure S7. Representative CV curves at 100 mV s^−1^ and comparison of electroactive surface area (ESA) change (%) for (a,b) CHI/Pt, and (c,d) CHI-thiomer/Pt brushes at different aptamer concentrations; Figure S8. (a) Representative Bode plot over the frequency range of 1–100,000 Hz (inset show exploded view over the frequency range from 1–5 Hz), and (b) representative Nyquist plots of impedance spectra for the CHI-thiomer/Pt brush sensor functionalized with 800 nM aptamer exposed to increasing concentration of *L. monocytogenes* in PBS.
