# Supplementary material for: One-Step Fabrication of Stimuli-Responsive Chitosan-Platinum Brushes for Listeria monocytogenes Detection
Source: Biosensors (Basel). 2021 Dec 13;11(12):511. doi: 10.3390/bios11120511 (PMC8699315; doi:10.3390/bios11120511)
Supplement: Supplementary file 1 [file biosensors-11-00511-s001.zip › biosensors-1450580-su updated.pdf]

Supplementary Materials

One-Step Fabrication of Stimuli-Responsive Chitosan-Platinum Brushes for *Listeria monocytogenes* Detection

Daniela A. Oliveira [1], Suleiman Althawab [1,2], Eric S. McLamore [3,*] and Carmen L. Gomes [1,4,*]

1 Department of Biological and Agricultural Engineering, Texas A&M University, College Station, TX 77843, USA; daoliveira@tamu.edu (D.A.O.); suleimanth@tamu.edu (S.A.)
2 Department of Nutrition and Food Science, Texas A&M University, College Station, TX 77843, USA
3 Department of Agricultural Sciences, Clemson University, Clemson, SC 26631, USA
4 Department of Mechanical Engineering, Iowa State University, Ames, IA 50011, USA
* Correspondence: emclamo@clemson.edu (E.S.M.); carmen@iastate.edu (C.L.G.); Tel.: +1-864-656-1001 (E.S.M.); +1-515-294-1138 (C.L.G.)

Materials and Methods

Thiomer Synthesis

Thiomers were synthesized by modifying chitosan (CHI) with thioglycolic acid (TGA) based on modification of Kast and Bernkop-Schnurch [1] using sulfo-NHS/EDC reaction to yield a high content of thiol termination on the resulting product. The modification was as follows: TGA carboxylate groups (-COOH) reacted to N-hydroxysulfosuccinimide (sulfo-NHS) in the presence of EDC (1-ethyl-3-[3-dimethylaminopropyl]carbodiimide hydrochloride), resulting in a semi-stable sulfo-NHS ester, which then reacted with primary amines (-NH2) on the chitosan to form amide crosslinks, resulting in a chitosan-TGA complex that is thiol-terminated.

First, a reaction was performed between TGA, EDC and sulfo-NHS (2:1:2.5 in molar concentration) in MES buffer containing sodium chloride at pH 5–6 for 30 min. The pH was then adjusted to 7.2–7.5 and a solution containing the chitosan (equal mass of TGA) was then added (doubling the volume). The pH was again adjusted to 6 and left to react under agitation for 2.5 h. After dialysis using 3.5 kDa tubing to eliminate the unbonded TGA and isolate the polymer conjugates, the eluent was freeze dried (–50 °C, –0.120 mBar, 48 h) in a Labconco FreeZone 6 unit (Labconco, Kansas City, MO, USA) to obtain the thiolated complex of chitosan-thioglycolic acid (CHI-thiomer).

The degree of thiolation was quantified using Ellman's Analysis [2]. Briefly, the reaction between the samples and the Ellman's reagent (5,5'-dithiobis-(2-nitrobenzoic acid)) was performed in a phosphate buffer containing 1mM EDTA (ethylenediamine tetraacetic acid) at pH 8. The absorbance of each sample was measured in a Genesys 10S spectrophotometer (Thermo Scientific, Waltham, MA) at 412 nm against a blank control and the thiol concentration calculated through the correlation with a standard curve of cysteine hydrochloride monohydrate. The result obtained was 40 ± 6 µM thiol groups/g of polymer sample, similar to Kast and Bernkop-Schnurch [1] that presented 38.23 µM thiol groups/g of polymer sample.

Sensor Biofunctionalization

An aptamer that targets the protein Internalin A on *Listeria monocytogenes* membrane known as (5′-ATC CAT GGG GCG GAG ATG AGG GGG AGG AGG GCG GGT ACC CGG TTG AT-3′) was purchased from GeneLink (Hawthrone, NY, USA). Upon receiving, the thiol terminated aptamers were reduced using dithiothreitol (DTT) and reconstituted in Tris EDTA (TE) buffer at pH 7.4, according to protocol provided

by the manufacturer (GeneLink). Decapped thiol-terminated aptamer with a C6 spacer at the 5′ end was loaded on the CHI/Pt (non-thiolated) and CHI-thiomer/Pt (thiolated) nanobrushes. For the CHI/Pt brush electrodes, the Sulfo-SMCC crosslinking reaction was used. Briefly, the first step was to individually immerse the CHI/Pt brush electrodes in 500 μL of the Sulfo-SMCC solution (5 mg Sulfo-SMCC dissolved in 100 μL filtered water then diluted in 9.9 mL PBS at pH 7.2) and allowed to react for 45 min at room temperature, shaking gently. Next, the maleimide-activated CHI/Pt brushes were placed in a PBS solution (pH 7.2) containing thiol-terminated aptamers (500, 1000 and 1500 nM) for 1 hour and 15 min, shaking gently [3]. Finally, the aptamer-CHI/Pt brushes were rinsed and stored in PBS until further use. For the CHI-thiomer/Pt brush electrodes, the aptamers were diluted in TE buffer and the electrodes submerged in this solution for two hours, shaking gently.

Results and Discussion

CHI/Pt Material Characterization

a)

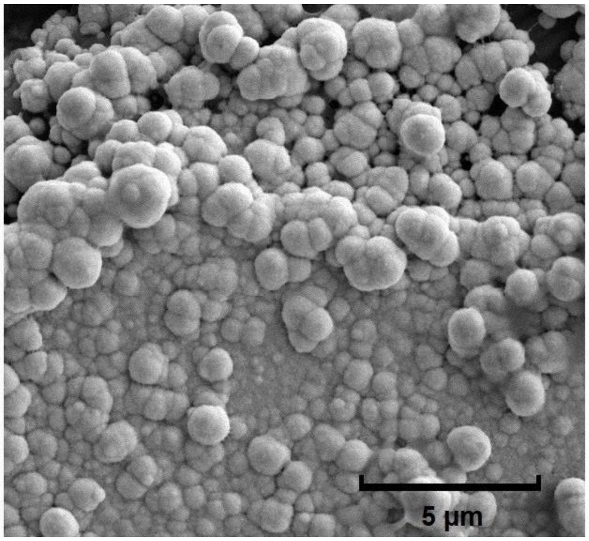

b)

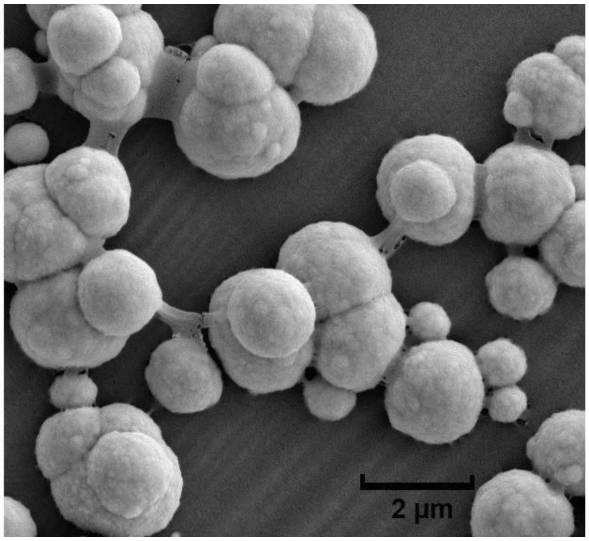

Figure S1. SEM images of CHI/Pt brushes at 10 kV. **a**) 10,100 and **b**) 15,000 times magnification showing a heterogeneous surface with non-uniform distribution of CHI/Pt brushes featuring spheroid structures.

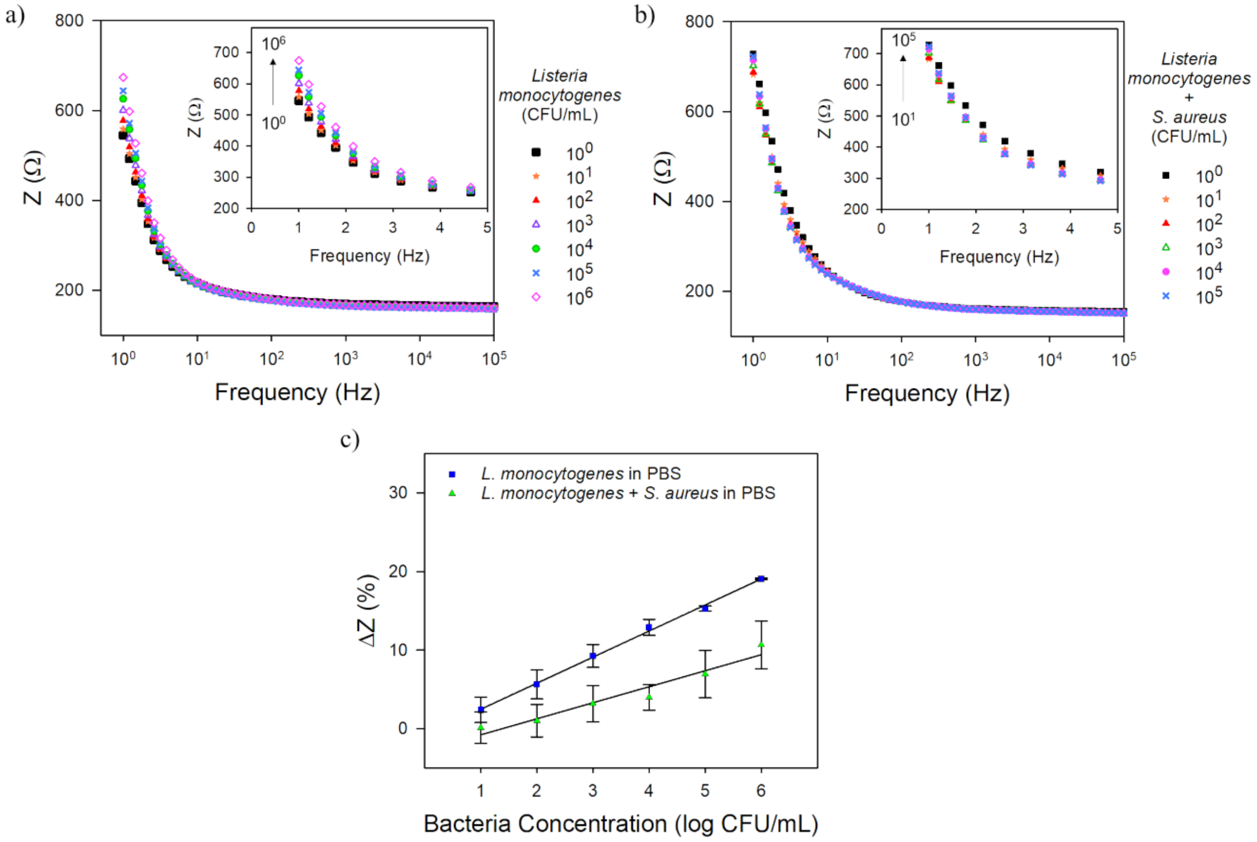

Figure S2. Representative Bode plot over the frequency range of 1–100,000 Hz (inset show exploded view over the frequency range from 1–5 Hz) for the CHI/Pt brush sensor without functionalization with aptamer exposed to **a**) L. monocytogenes in PBS, **b**) L. monocytogenes and S. aureus in PBS, and **c**) calibration curves (impedance change at 1 Hz vs. log bacteria concentration). All data represents the average of three repetitions. Error bars represent the standard deviation.

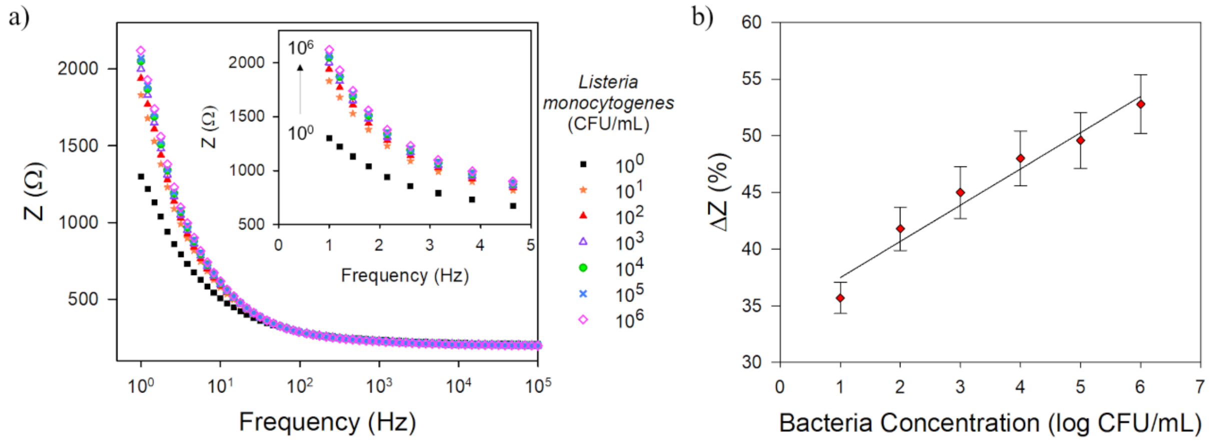

Figure S3. Representative Bode plot over the frequency range of 1–100,000 Hz (inset show exploded view over the frequency range from 1–5 Hz) for the CHI/Pt brush sensor without functionalization with aptamer exposed to a) L. monocytogenes in chicken broth, and b) calibration curve (impedance change at 1 Hz vs. log bacteria concentration). All data represents the average of three repetitions. Error bars represent the standard deviation.

Table S1. Performance of the CHI/Pt brush biosensor without functionalization with aptamer when exposed to Listeria monocytogenes in different media.

**Test Medium**	**Detection Range (CFU/mL)***
PBS	3.1 – 10^6
PBS + *S. aureus*	11 – 10^5
Chicken broth	22 – 10^6

* lowest value corresponds to the lower limit of detection (LOD).

CHI-thiomer/Pt Material Characterization

The main differences on the spectra of the CHI-thiomer/Pt (Figure S1) from the CHI/Pt (Figure 2 on the main manuscript) brush depositions are: 1) the presence of the S 2p peak and 2) the Pt 4f is deconvoluted into two doublets at 71, 72, 74.4, and 75.3 eV, which is consistent with literature reports for unbound Pt^0 and S-bonded Pt [4–6]. This was expected as platinum has tremendous affinity for binding to sulfur donors compared to nitrogen donor ligands [7].

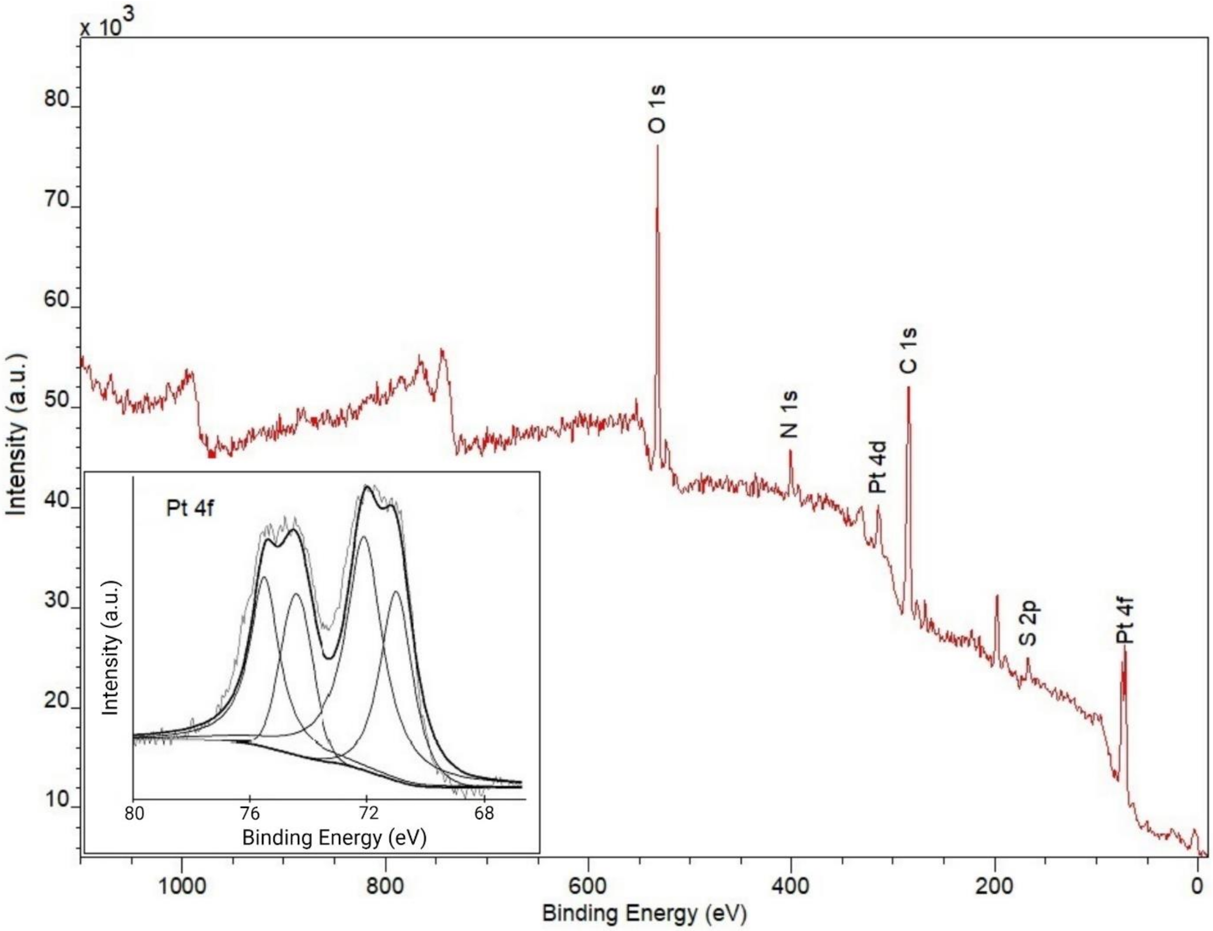

Figure S4. X-ray photoelectron spectroscopy (XPS) survey spectrum of the CHI/Pt brush deposition. Inset shows the Pt 4f spectrum indicating the presence of S 2 p peak unbound Pt0 and S-bonded Pt.

Figure S5 shows the optimized CHI-thiomer/Pt brush deposition (60 cycles / 6 V / 0.15% CHI-thiomer/Pt). The electrodeposition of CHI-thiomer/Pt brush led to heterogenous spatial distribution and non-uniform brush sizes distribution on the electrode surface. The SEM images indicate that there is a Pt layer covering the entire electrode and some clusters of CHI-thiomer/Pt brushes dispersed over it forming a network of spherical particles ranging from 100 to 1000 nm.

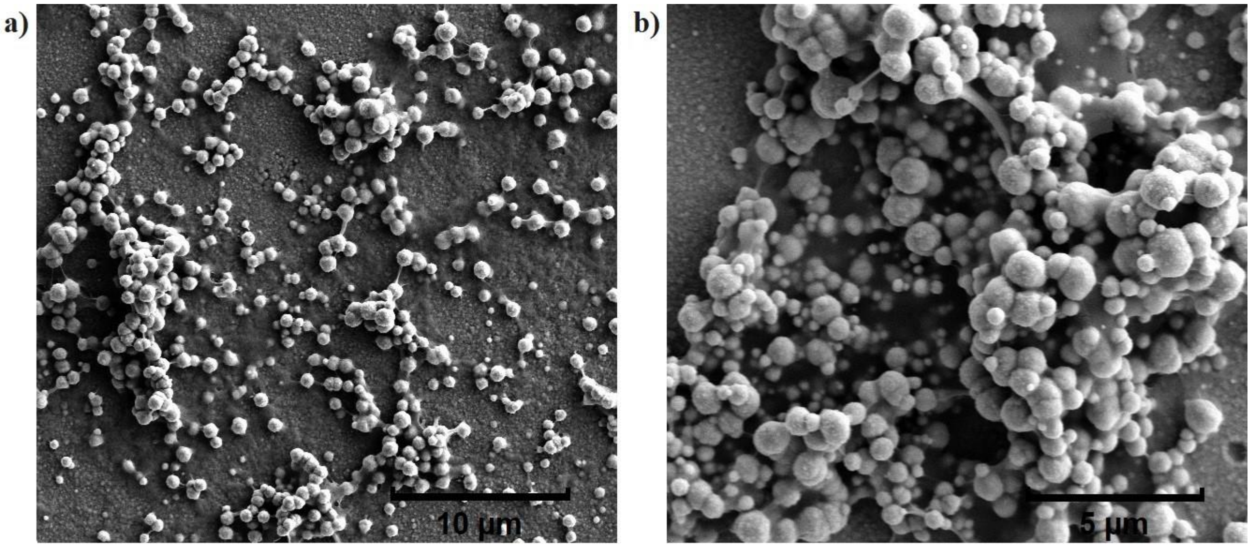

Figure S5. SEM images of CHI-thiomer/Pt brushes at 10 kV and **a**) 5,100 and **b**) 10,100 times magnification indicate a non-homogenous and dispersed brush distribution with varying brush sizes resembling a network of spherical particles with diameters between 100 and 1000 nm.

Electrochemical Characterization and Actuation of CHI-thiomer/Pt Nanobrushes

When applying 60 cycles / 6V / 0.15% CHI-thiomer/Pt for the deposition, the increase in ESA was of 12 times (0.36 ± 0.01 cm^2) compared to bare Pt/Ir electrodes (Figure S6a). The hypothesis behind the modification of the chitosan with TGA was that the incorporation of a thiol termination would deposit chitosan brushes embedded with Pt nanoparticles that was expected to result in a more significant increase on the ESA than observed here when compared to the non-modified CHI/Pt brush deposition. Even though ESA values increased by 12 times with CHI-thiomer/Pt, the non-modified CHI/Pt deposition showed an ESA increase of 11 times without the need for chemical modification as well as a more homogeneous brush deposition onto the electrodes (Figures 3, 4 and S1) which will later demonstrate enhanced sensing performance. The actuation tests performed using different redox probes with the CHI-thiomer/Pt brush electrodes (Figure S6b) show that the modification of chitosan with thioglycolic acid did not affect the pH stimuli property of chitosan exhibiting the same behavior shown in Figure 5 for the CHI/Pt brush electrodes. Previous studies have shown that the use of a lower molecular weight chitosan resulted in higher thiolation efficiency compared to higher molecular weight; however, it also showed a reduced response to pH changes in the environment [8–10], thus resulting in less actuation.

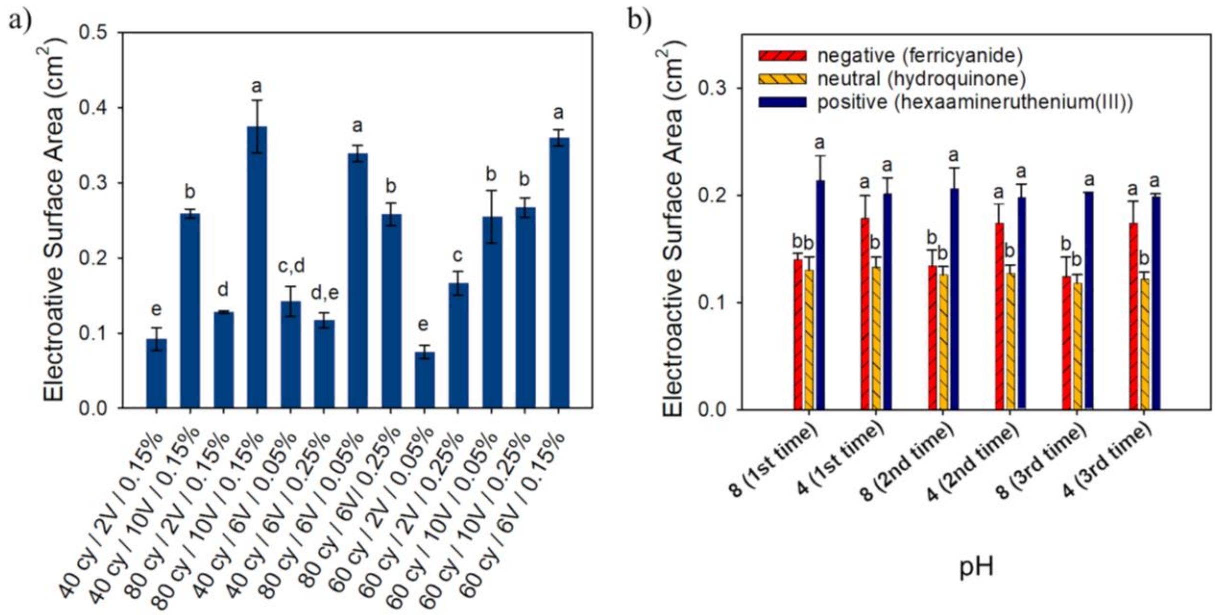

Figure S6. Electrochemical characterization of CHI-thiomer/Pt brushes. (**a**) Average electroactive surface area (ESA) for various electrode modifications with different number of cycles (cy), voltages (V) and CHI-thiomer concentration (wt.%) using 4 mM K4FeCN6 as redox probe. (**b**) Electrostatic interactions during CHI-thiomer/Pt brush actuation for various redox probes: a negatively charged probe ($KFeCN_6^{3-}$), a neutral probe ($C_6H_4(OH)_2$), and a positively charged probe ($Ru(NH_3)_6^{3+}$. The average electroactive surface area (ESA) is shown for each redox probe under repeated actuation at pH 4 and pH 8 (n = 3). All data represent the average of three replicates and error bars represent the standard deviation of the arithmetic mean; letters denote significantly different means ($p < 0.05$).

Aptamer Loading

The functionalization of aptamers onto CHI/Pt and CHI-thiomer/Pt brushes was optimized using CV. As shown in Figure S5, the aptamer attachment caused a decrease in ESA values due to steric hindrance, which was also observed by Hills et al. [11]. This could be expected as the aptamer used is a nucleic acid sequence and it has been shown that DNA can act as an insulator [12–14]. The CHI-thiomer/Pt brushes (Figures S7c and S7d) presented similar ($p > 0.05$) ESA percentage change independent of the aptamer concentration loaded and 800 nM was chosen for further experiments with bacteria. For the CHI/Pt brushes functionalized with aptamers (Figures S7a and S7b), the ones loaded with 1000 nM presented the highest value in ESA change, despite of not being significantly different ($p > 0.05$) from the ones with 1500 nM. Hence, the aptamer concentration of 1000 nM was chosen for better comparison with previous results from our group when working with chitosan.

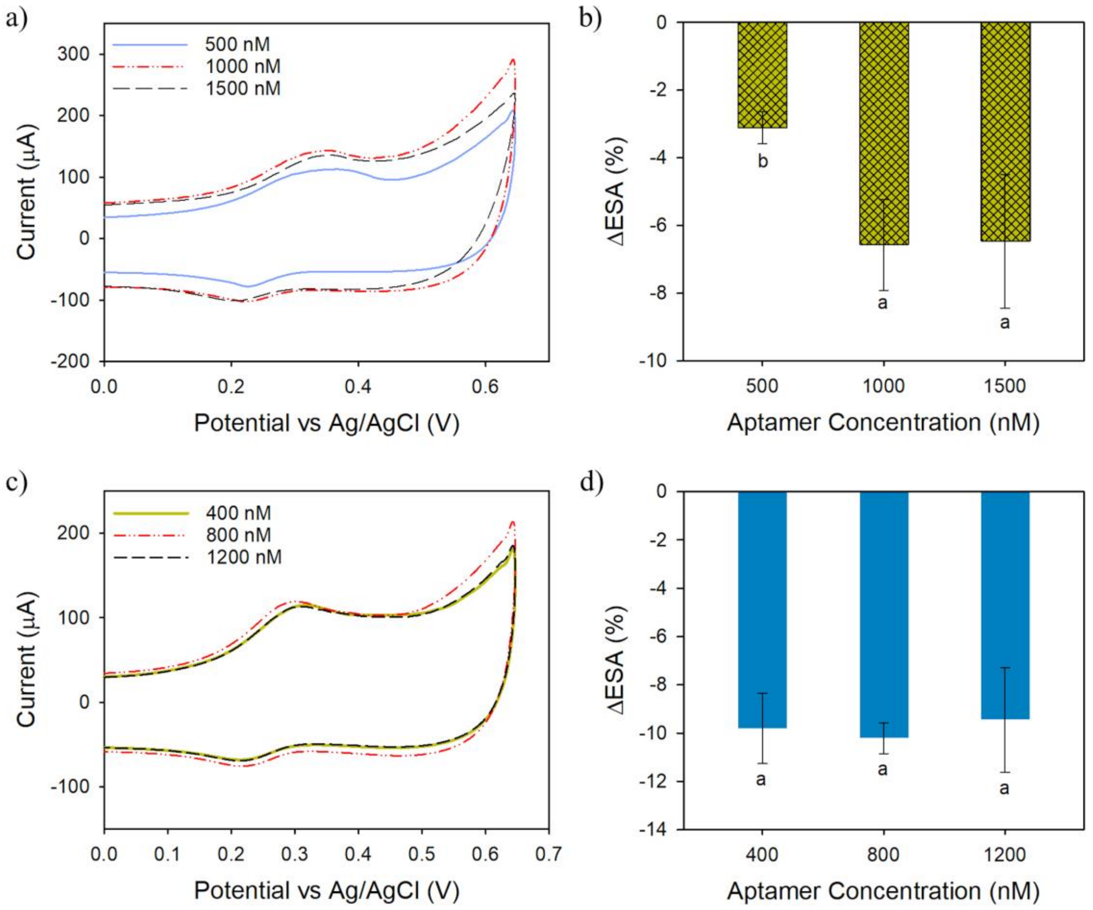

Figure S7. Representative CV curves at 100 mV s^{-1} and comparison of electroactive surface area (ESA) change (%) for (**a**, **b**) CHI/Pt, and (**c**, **d**) CHI-thiomer/Pt brushes at different aptamer concentrations. Curves represent the average of three replications. Bars denoted by different letters are significantly different from each other ($p < 0.05$). Error bars represent the standard deviation.

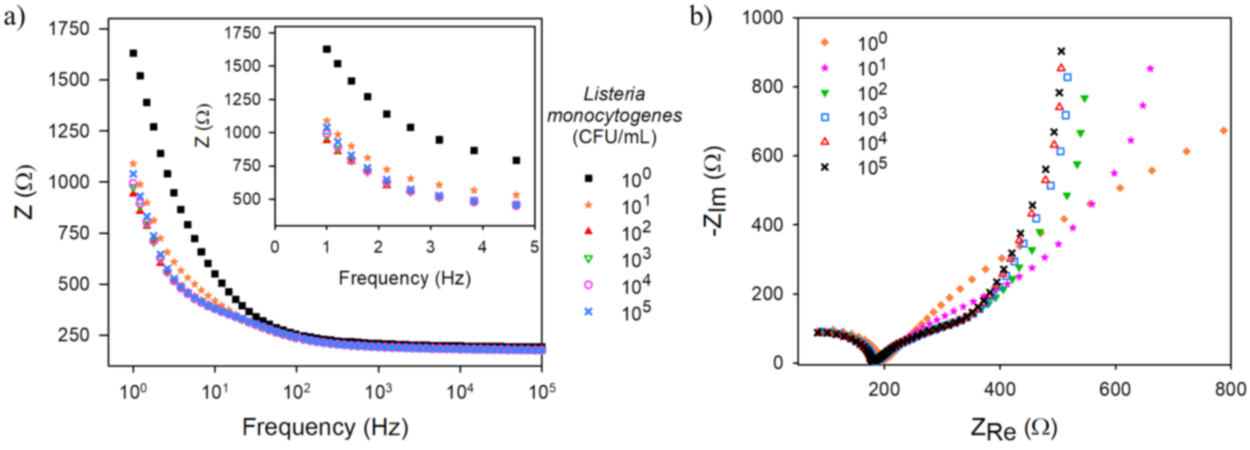

Figure S8. **a**) Representative Bode plot over the frequency range of 1–100,000 Hz (inset show exploded view over the frequency range from 1–5 Hz), and **b**) representative Nyquist plots of impedance spectra for the CHI-thiomer/Pt brush sensor functionalized with 800 nM aptamer exposed to increasing concentration of L. monocytogenes in PBS. All data represents the average of three repetitions.

References

1. Kast, C.E.; Bernkop-Schnurch, A. Thiolated Polymers–Thiomers: Development and in Vitro Evaluation of Chitosan-Thioglycolic Acid Conjugates. *Biomaterials* **2001**, *22*, 2345–2352.
2. Thermo Scientific Ellman's Reagent **2011**.
3. Balamurugan, S.; Obubuafo, A.; Soper, S.A.; Spivak, D.A. Surface Immobilization Methods for Aptamer Diagnostic Applications. *Anal. Bioanal. Chem*. **2008**, *390*, 1009–1021, doi:10.1007/s00216-007-1587-2.
4. Yang, B.; Agrios, A.G. Attachment of Pt Nanoparticles to a Metal Oxide Surface Using a Thiol–Carboxyl Bifunctional Molecule OH OH OH. *J. Colloid Interface Sci*. **2018**, *513*, 464–469, doi:10.1016/j.jcis.2017.11.058.
5. Kwon, K.; Jin, S.; Pak, C.; Chang, H.; Joo, S.H.; Lee, H.I.; Kim, J.H.; Kim, J.M. Enhancement of Electrochemical Stability and Catalytic Activity of Pt Nanoparticles via Strong Metal-Support Interaction with Sulfur-Containing Ordered Mesoporous Carbon. *Catal. Today* **2011**, *164*, 186–189.
6. Romanchenko, A.; Likhatski, M.; Mikhlin, Y. X-Ray Photoelectron Spectroscopy (XPS) Study of the Products Formed on Sulfide Minerals Upon the Interaction with Aqueous Platinum (IV) Chloride Complexes. *Minerals* **2018**, *8*, 578, doi:10.3390/min8120578.
7. Volckova, E.; Dudones, L.P.; Bose, R.N. HPLC Determination of Binding of Cisplatin to DNA in the Presence of Biological Thiols : Implications of Dominant Platinum-Thiol Binding to Its Anticancer Action. *Pharm. Res.* **2002**, *19*, 124–131.
8. Yousefpour, P.; Atyabi, F.; Dinarvand, R.; Vasheghani-Farahani, E. Preparation and Comparison of Chitosan Nanoparticles with Different Degrees of Glutathione Thiolation. *Daru. J. Fac. Pharm*. **2011**, *19*, 367–375.
9. Andreas Bernkop-Schnürch; Weithaler, A.; Karin Albrecht; Greimel, A. Thiomers: Preparation and in Vitro Evaluation of a Mucoadhesive Nanoparticulate Drug Delivery System. *Int. J. Pharm*. **2006**, *317*, 76–81.
10. Chauhan, K.; Singh, P.; Singhal, R.K. New Chitosan–Thiomer: An Efficient Colorimetric Sensor and E Ff Ective Sorbent for Mercury at Ultralow Concentration. *ACS Appl. Mater. Interfaces* **2015**, *7*, 26069–26078, doi:10.1021/acsami.5b06078.
11. Hills, K.D.; Oliveira, D.A.; Cavallaro, N.D.; Gomes, C.L.; McLamore, E.S. Actuation of Chitosan-Aptamer Nanobrush Borders for Pathogen Sensing. *Analyst* **2018**, *143*, 1650–1661, doi:10.1039/c7an02039b.
12. Zhang, Y.; Austin, R.H.; Kraeft, J.; Cox, E.C.; Ong, N.P. Insulating Behavior of λ-DNA on the Micron Scale. *Phys. Rev. Lett*. **2002**, *89*, 2–5, doi:10.1103/PhysRevLett.89.198102.
13. Gómez-Navarro, C.; Moreno-Herrero, F.; Pablo, P.J. de; Colchero, J.; Gómez-Herrero, J.; Baró, A.M. Contactless

Experiments on Individual DNA Molecules Show No Evidence for Molecular Wire Behavior. *Proc. Natl. Acad. Sci.* **2002**, *99*, 8484–8487.
14. Bockrath, M.; Markovic, N.; Shepard, A.; Tinkham, M.; Gurevich, L.; Kouwenhoven, L.P.; Wu, M.W.; Sohn, L.L. Scanned Conductance Microscopy of Carbon Nanotubes and λ -DNA. *Nano Lett*. **2002**, *2*, 187–190.
